# The Epidemiology and Clinical Manifestations of Takayasu Arteritis: A Descriptive Study of Case Reports

**DOI:** 10.7759/cureus.17998

**Published:** 2021-09-15

**Authors:** Dina Alnabwani, Palak Patel, Priyaranjan Kata, Vraj Patel, Arthur Okere, Pramil Cheriyath

**Affiliations:** 1 Internal Medicine, Hackensack Meridian Health Ocean Medical Center, Brick, USA; 2 Cardiology, Ocean Medical Center, Brick, USA

**Keywords:** takayasu disease, claudication, pulseless disease, granulomatous inflammatory vasculitis, transmural fibrous thickening of the arterial walls

## Abstract

Takayasu's arteritis is a rare form of chronic inflammatory disorder involving large vessels, with an unclear etiology. Common early signs and symptoms are weakness, malaise, and fever. Takayasu's arteritis mainly involves the aorta and its branches as well as the subclavian and carotid arteries. While radiologic methods can identify diseased vessels, they can't tell the difference between active and chronic lesions. This study reviews the characteristics of Takayasu's arteritis to identify any possible changes in the prevalence of symptoms of the disease.

We conducted a literature review of case reports on Takayasu arteritis from PubMed and Google Scholar. Variables of interest were age, gender, symptoms, blood pressure (BP) measurement, diminished pulses, and radiological findings. Data were transferred to an Excel spreadsheet (Microsoft Corporation, Redmond, WA), and mean, median, and standard deviation, frequencies, and proportions were calculated using R version 1.1.456 (RStudio: Integrated Development for *R*. RStudio, PBC, Boston, MA).

There were 43 cases, and females accounted for 88.3% of the presentations. The average age was 25 years, SD 12.5 years. Fever was the most frequent symptom (20.93%), followed by chest pain (13.95%), claudication (13.95%), and headache (13.95%). Less frequent complaints included shortness of breath (11.62%), weight loss (9.30%), syncope (6.98%), and night sweats (4.65%). On the right side, the average BP was 142/87 mmHg, and the left-sided finding averaged 115/72 mmHg. Decreased pulses were primarily seen in the radial artery with 15 cases. Radiological findings showed narrowing of the vessels in the following order: aorta (22), carotid (11), renal (10), subclavian (9), celiac (2), mesenteric (2), axillary (2), and tibial (1).

The characteristics of Takayasu's arteritis were analyzed in this study. It identified several findings, ranging from fever symptoms to the signs of claudication, as well as the involvement of major vessels, such as the aorta and its branches, and a summary of radiological findings. This depicts the picture of Takayasu's arteritis and what physicians should expect when dealing with the disease.

## Introduction and background

Takayasu arteritis (TA), often known as pulseless disease, is chronic inflammatory arteritis characterized by damage to the medium and large arteries as well as their branches. The aorta and its primary branches, particularly the renal, carotid, and subclavian arteries, are commonly affected, resulting in stenosis, occlusions, or aneurysmal degeneration of these large vessels [1]. Despite the fact that the disease is widespread throughout the world, the Asian population is thought to be far more affected. In Japan, the greatest known prevalence of Takayasu arteritis was assessed to be 40 per million while in the United States, the lowest known frequency was reported to be 0.9 per million [2]. Although not all patients follow this pattern, a two-stage continuum has been proposed, with a “pre-pulseless” period marked by non-specific inflammatory features, followed by a chronic phase marked by the onset of vascular insufficiency and, in some cases, flares [3]. We performed a descriptive study of cases of Takayasu arteritis to examine the epidemiology, clinical manifestations, and management.

## Review

Methods

Eligibility Criteria

All case reports published on the Internet under the name Takayasu arteritis.

Search Strategies

A systematic search of Takayasu arteritis case reports utilizing Google, Yahoo, Medscape, Scopes, and Cochrane, and scientific databases such as Google Scholar and PubMed.

Data Collection Process and Data Items

Using standardized data extraction forms, data were extracted independently by two authors. We collected characteristics like age, gender, initial symptom presentation, laboratory values, vital signs, diagnostic imaging, and echocardiography results on an Excel sheet (Microsoft Corporation, Redmond, WA), and these variables were analyzed.

Statistical Analysis

Patient demographic characteristics, disease manifestations, and causes were summarized descriptively and analyzed using R version 1.1.456 (RStudio: Integrated Development for R. RStudio, PBC, Boston, MA).

Results

We reviewed 43 case reports available online and gathered all the data regarding the characteristics and presentation of initial symptoms. Table 1 describes the proportion of initial clinical presentations of TA. 

**Table 1:** Characteristics and presentation of initial symptoms and signs ESR: erythrocyte sedimentation rate; CRP: c-reactive protein

Parameter	Value (±SD)
Age	25 (12.50)
Males	11.62%
Females	38%
Fever	20.93%
Chest pain	13.95%
Claudication symptoms	13.95%
Headache	13.95%
Shortness of breath	11.62%
Weight loss	9.30%
Syncope	6.98%
Night sweat	4.65%
Blood pressure	
Right systolic BP	142.37 mmHg (±42.78 mmHg)
Right diastolic BP	87.30 mmHg (±29.67 mmHg)
Left systolic BP	115 mmHg (±45.15 mmHg)
Left diastolic BP	72.4 mmHg (±27.21 mmHg)
Laboratory Value	
ESR	63.58 (±34.68)
CRP	38.78 (±57.40)
Echocardiography	
Ejection fraction	38.79% (±57.40%)
Mitral regurgitation	11.63%
Aortic regurgitation	4.65%
Tricuspid regurgitation	4.65%
Left ventricular hypertrophy	2.32%

The average age of the population is 25 years, with a standard deviation of 12.5 years. Females accounted for 88.3% of the cases while males accounted for 11.6 %. The most common symptom of Takayasu arteritis was fever (20.93%), followed by chest discomfort (13.95%), claudication (13.95%), and headache (13.95%). Shortness of breath was reported in 11.62% of cases, weight loss in 9.30% of cases, syncope in 6.98% of cases, and night sweats in 4.65% of cases. Out of all the collected data, 15 cases reported absent or decreased pulse in the radial artery with five cases of brachial pulses. A case of an absent pulse of the subclavian, carotid, femoral, and dorsal arteries was also reported. In 6.97%, there was a bruit, and in 6.98 % of cases, there was a murmur. We noticed the following vessels implicated on imaging stated in case reports (Table 2).

**Table 2:** Vessels implicated in imaging stated in case reports

Narrowing of artery in MRI or CT scan	Value (number)
Aorta and its branches	22
Carotid	11
Renal	10
Subclavian	9
Celiac	2
Mesenteric	2
Axillary	2
Tibial	1

Discussion

Takayasu arteritis is a difficult condition to deal with. Early identification of this disease is difficult and necessitates clinical suspicion and vigilance. It is a granulomatous inflammatory vasculitis of the medium and large arteries that results in transmural fibrous thickening of the arterial walls, causing vascular blockages and ischemic changes [1]. While all major arteries may be impaired, the ascending/descending aorta, subclavian arteries, and extracranial arteries like the carotids are the most commonly affected vessels (60-90%) [4].

The etiopathogenesis of the disease has yet to be determined. Infections, autoimmune, and genetic factors have all been investigated as etiologic causes. There has been speculation that there is a link between TA and tuberculosis (TB). Both illnesses cause arterial wall changes that are similar in appearance such as chronic inflammatory lesions, and, in rare cases, granulomas of large and medium vessels [5]. Despite the relationship with tuberculosis and parallels in granulomatous lesions, the role of Mycobacterium tuberculosis in the genesis of TA is unknown. Cross-reactions between Mycobacterium and human heat-shock protein (HSP) have been proposed to play a significant role in previous research [6-7]. Viral infection is being investigated as a possible cause of vasculitis since vascular lesions are comparable to those seen in animals with viral infections [8]. Autoimmune aortitis (syphilis, TB, lupus, rheumatoid arthritis, spondyloarthropathies, Behçet's illness, Kawasaki illness, and giant cell arteritis); developmental disorders (coarctation of the aorta and Marfan syndrome); and other aortic diseases (ergotism and neurofibromatosis) are other causes of large vessel vasculitis [3].

It's now assumed that an unknown trigger causes the 65kDa heat-shock protein to be expressed in aortic tissue, which subsequently causes the major histocompatibility class I chain-related A (MICA) to be expressed on vascular cells. T cells and NK cells with NKG2D receptors recognize MICA on vascular smooth muscle cells and release perforin, resulting in acute vascular inflammation. Th1 lymphocytes release interferon-, which causes giant cells to form and activate macrophages by releasing vascular endothelial growth factor (VEGF) and platelet-derived growth factor (PDGF), which cause neovascularization and smooth muscle migration and intimal proliferation, respectively. The IL-23 microenvironment promotes the recruitment of infiltrating neutrophils by Th17 cells, which adds to vascular lesions. Dendritic cells and B lymphocytes can work together to stimulate the production of anti-endothelial cell autoantibodies, resulting in complement-dependent cytotoxicity of endothelial cells, however, this is still debatable [9]. The great majority of Takayasu arteritis patients suffer from a widespread illness. In the lack of specific therapy, the natural history of any condition can only be elucidated by observing patients. Ishikawa divided patients into specialized groups depending on the nature and severity of their illnesses (Table 3) [10].

**Table 3:** Ishikawa clinical classification of Takayasu arteritis

Groups	Clinical features
Group I	Uncomplicated disease, with or without pulmonary artery involvement
Group IIA	Mild/moderate single complication together with uncomplicated disease
Group IIB	Severe single complication together with uncomplicated disease
Group III	Two or more complications together with uncomplicated disease

Based on the more prevalent symptoms, the American College of Rheumatology (ACR) adopted precise diagnostic guidelines for Takayasu's arteritis in 1990. At least three of the six criteria must be met to diagnose Takayasu arteritis (Table 4) [11].

**Table 4:** American College of Rheumatology (ACR) diagnostic guidelines for Takayasu's arteritis in 1990

ACR Criteria	Definition
Age at disease onset ≤40 years	Development of symptoms or findings related to Takayasu arteritis at age ≤40 years
Claudication of extremities	Development and worsening of fatigue and discomfort in muscles of 1 or more extremity while in use, especially the upper extremities
Decreased brachial artery pulse	Decreased pulsation of 1 or both brachial arteries
Blood pressure difference >10 mm Hg	Difference of >10 mm Hg in systolic blood pressure between arms
Bruit over subclavian arteries or aorta	Bruit audible on auscultation over 1 or both subclavian arteries or abdominal aorta
Arteriogram abnormality	Arteriographic narrowing or occlusion of the entire aorta, its primary branches, or large arteries in the proximal upper or lower extremities, not caused by arteriosclerosis, fibromuscular dysplasia, or similar causes; changes usually focal or segmental

Takayasu arteritis has a wide range of clinical symptoms, ranging from asymptomatic disease induced by impalpable pulse or bruits to serious neurological impairment. Non-specific symptoms include fever, night sweats, tiredness, weight loss, arthralgia, myalgia, and mild anemia [12]. As the inflammation progresses, more stenoses form and distinct characteristics appear, owing to the development of collateral circulation. The most prevalent type of lesion is stenosis, which is frequently bilateral [13-14]. As the condition advances, the symptoms and effects become increasingly severe, leading to complications. Takayasu retinopathy, secondary hypertension, aortic regurgitation, and aneurysm formation were the four most common sequelae observed in Takayasu patients, each of which was categorized as mild/moderate or severe at the time of diagnosis [10]. Clinical manifestations, laboratory values, mainly inflammatory markers, and diagnostic imaging are all used to diagnose Takayasu arteritis. Angiography is still the gold standard for diagnostic and treatment planning. The use of angiography to examine the pulmonary vasculature is not routinely suggested; it is reserved for individuals who have symptoms of pulmonary hypertension. The use of Doppler ultrasound to assess vascular wall inflammation is a non-invasive approach. Histological diagnosis is frequently impractical due to the vessels involved, and histological testing is limited to those patients undergoing revascularization procedures [14].

For medical management, steroids are the mainstay of treatment for Takayasu arteritis. Steroids have a 50% response rate, and methotrexate, which has a 50% response rate, are the best evidence-based therapy. Methotrexate's use as a steroid-sparing medication is both reasonable and safe. Twenty-five percent of patients with active disease will not respond to existing treatments, and it is important to avoid exposing these patients to the risks of extended immunosuppression in the lack of other options. Another key medical issue is the use of steroids, particularly in patients with critical hypertension, because hypertension is difficult to manage and is exacerbated by the use of steroids, which have fluid-retaining adverse effects [15].

Hypertension with critical renal artery stenosis, extremity claudication limiting daily activities, cerebrovascular ischemia or critical stenoses of three or more cerebral vessels, moderate aortic regurgitation, and cardiac ischemia with confirmed coronary artery involvement are all indications for surgery [14]. In general, surgery is recommended to avoid co-morbidities, which include restenosis, anastomotic failure, thrombosis, hemorrhage, and infection at a time when the disease is silent [14]. Surgery for aortic arch and splanchnic disease may be unnecessary due to substantial collateral development around the stenosis [15]. However, critical stenoses should be corrected to prevent stroke. Renal artery involvement is best treated with percutaneous transluminal angioplasty [16].

There is a classification depending on vessel involvement that can aid in planning surgery and may provide a prognosis (Table 5) [17].

**Table 5:** New angiographic classification of Takayasu arteritis

Type	Vessel Involvement
Type I	Branches from the aortic arch
Type IIa	Ascending aorta, aortic arch and its branches
Type IIb	Ascending aorta, aortic arch and its branches, thoracic descending aorta
Type III	Thoracic descending aorta, abdominal aorta, and/or renal arteries
Type IV	Abdominal aorta and/or renal arteries
Type V	Combined features of types IIb and IV

Takayasu arteritis is a relatively uncommon condition that can manifest itself in subtle and insidious ways. Nonetheless, prompt diagnosis is essential since failing to treat vascular inflammation in a timely manner might have serious consequences for the patient. There are two categories of symptoms that can alert a clinician to the presence of Takayasu arteritis: A systemically unwell patient with nonspecific laboratory results pointing to inflammatory disease. Cardiovascular features, such as claudication of the limbs, hypertension, arterial pain, and distal cutaneous hypothermia, especially in a young patient or one who does not have atherosclerosis risk factors [18].

## Conclusions

The characteristics of Takayasu's arteritis were investigated in this study. Several physical findings and their corresponding values, as well as a summary of radiological findings, have been listed. Based on the review and analysis of all the case reports on Takayasu arteritis, we observed that Takayasu arteritis is most commonly seen in females, with an average onset age of 13-38 years. The majority of patients present with a wide range of symptoms, from fever to signs of claudication and the associated symptoms. The results showed the difference in blood pressure measurements of two distinct limbs with a decreased or absent pulse, primarily radial pulse. Takayasu could be a differential in symptomatic patients with high ESR and CRP levels. The main entity for diagnosis is imaging, which depicts stenosis of major vessels and other conditions. Because of the clot formation, there may be some changes in heart function as well as related consequences. This paints a picture of the landscape of Takayasu’s arteritis and what physicians should expect when dealing with the disease.
